# Development of optimal indoor air disinfection and ventilation protocols for airborne infectious diseases

**DOI:** 10.1371/journal.pone.0311274

**Published:** 2024-10-01

**Authors:** Jooyeon Park, Kyoung Hwa Lee, Young Goo Song, Hyungmin Park, Kwang Suk Lee

**Affiliations:** 1 Department of Mechanical Engineering, Seoul National University, Seoul, Korea; 2 Department of Internal Medicine, Division of Infectious Diseases, Gangnam Severance Hospital, Yonsei University College of Medicine, Seoul, Korea; 3 Institute of Advanced Machines and Design, Seoul National University, Seoul, Korea; 4 Department of Urology, Gangnam Severance Hospital, Yonsei University College of Medicine, Seoul, Korea; Satyawati College, University of Delhi, INDIA

## Abstract

Since the COVID-19 pandemic, there has been persistent emphasis on the importance of indoor air disinfection and ventilation in isolation units in the hospital environment. Nevertheless, no optimal and concrete disinfection protocol has been proposed to inactivate the viruses as quickly as possible. In this study, we experimentally evaluated various ventilation and disinfection protocols based on the combination of negative-pressure ventilation, ultraviolet (UV) light illumination, and Hypochlorous acid (HOCl) spray against three active virus species in a 3.5 cubic meters isolation unit. This small-size unit has gained attention during the pandemic due to the high demand for compact mobile laboratory systems capable of rapid disease diagnosis. In accordance with the WHO laboratory biosafety guidance, which states that all enclosed units where diagnostic work is conducted must ensure proper ventilation and disinfection activities, we aim to propose virus removal protocols for units compact enough to be installed within a van or deployed outdoor. The results confirmed the superiority (in terms of virus removal rate and time required) of the virus removal methods in the order of UV light, ventilation, and HOCl spray. Ultimately, we propose two optimal protocols: (i) UV light alone for three minutes, and (ii) UV light with ventilation for three minutes, followed by one-minute ventilation only. The time span of three minutes in the latter protocol is based on the clinical practice such that the medical staffs have a sufficient time to process the samples taken in transition to next patient to care.

## 1. Introduction

Over the past few decades, we have encountered a number of respiratory infectious diseases and been forced to realize that effective ventilation in indoor medical environments is a critical factor for infection prevention and control [1]. Thus, many previous studies have performed experiments and numerical simulations to regulate indoor airflows in an effort to prevent the spread of contagious pathogens [2–5]. Yang *et al*. [2] numerically determined the efficiency of mechanical displacement ventilation in reducing CO_2_ concentration in a simple cubic chamber while varying the ventilation rate. Unlike the general expectation, it was shown that the elimination rate of infectious pathogens does not increase with increasing flow rates. If the ventilation rate is too high, strong inflow will be induced to break down the pollutant stratification near the ceiling of the chamber, which is the key mechanism of displacement ventilation. Weiland *et al*. [3] experimentally compared the effects of room pressure and air exchange rate on particle release out of the room in different compartment configurations (distance between the door and ventilation system) of the hospital. They recommended that the healthcare workers involved in the aerosol-generating procedures maintain the negative pressure regardless of the ventilation rate. The CDC also supports and instructs the maintenance of negative pressure in patient rooms to prevent a cross-infection [6].

In hospital environments such as clinics, emergency rooms, nursing homes, long-term care facilities, and so on, the probability of airborne transmission is extremely high because the concentration of contagious pathogens is much higher than in other indoor environments [7]. Thus, more effective and rigorous virus removal protocols should be established based on the physical grounds rather than the experiences from the fields. Several respiratory infectious disease outbreaks in hospitals have been reported, and this is emerging as an important health problem in hospital-associated infections. Ultimately, this contributes to the worldwide spread of the diseases through airborne transmission [3,8–10]. Furthermore, massive testing is desperately necessary in a pandemic situation to quickly identify the pathogen and prevent widespread infection. The contagious pathogens remaining inside the medical facilities must be completely disinfected as quickly as possible for efficient turnover.

In our previous research, we systematically conducted a series of numerical investigations to uncover the optimized ventilation mechanism for fast removal of infectious pathogen-laden aerosols in the negative-pressure unit (targeting the compact isolated disease screening/clinic room) [11]. It was demonstrated that the most critical aspect of designing the ventilation system is to effectively guide the patient’s respiratory flow directly to the outlet of the negative-pressure unit without causing dispersion (or mixing with the inflow), which is irrelevant to the magnitude of the flow rate. Based on this mechanism, we developed a displacement ventilation configuration with the inlet and outlet installed as a thin slit near the floor and on the top of the unit, respectively. Other investigations have also reported that the displacement type is more energy-efficient from the perspective of virus removal, and health organizations have developed displacement strategy guidelines and recommendations for engineers [1,12–14].

In this follow-up study, based on the proposed design, we built a full-sized isolated unit for infectious disease screening, which is used to identify the most effective virus removal procedures and protocols. To achieve perfectly clean air in the unit within a short time duration, determined based on the actual clinical settings in the fields, we experimentally evaluated the virus inactivation and removal performance of various configurations combined with typical methods used in clinical practice: ultraviolet (UV) light illumination, hypochlorous acid (HOCl) spray, and negative-pressure ventilation. HOCl is known to be an effective disinfectant against the contagious virus at an oxidative titratable chlorine (Cl) concentration of 45–60 ppm in healthcare settings [15]. UV radiation has also been employed for disinfecting water, air, and organic matters [16]. This study aims to evaluate the actual elimination rate of three contagious respiratory viruses using various sterilization methods. Our main objective is to completely eliminate infectious viruses within the isolated unit in the shortest possible time after a patient has left and before the next patient enters. For this reason, the by-products of UV-C exposure such as skin reddening (erythema) and eye inflammation (photokeratitis) [17] are not considered in this study.

## 2. Method

### Study design (sterile chamber isolation unit)

The evaluations were performed in two stages. In the first stage, the virus removal and inactivation performance of each element of ventilation, UV light, and HOCl spray was compared, respectively, focusing on the shortest time required. In the second stage, the optimal indoor virus removal strategies were drawn, which inactivate or eliminate more than 99% of viruses in the fastest time, combining two elements verified in the first stage (the hybrid protocol).

**Fig 1:**
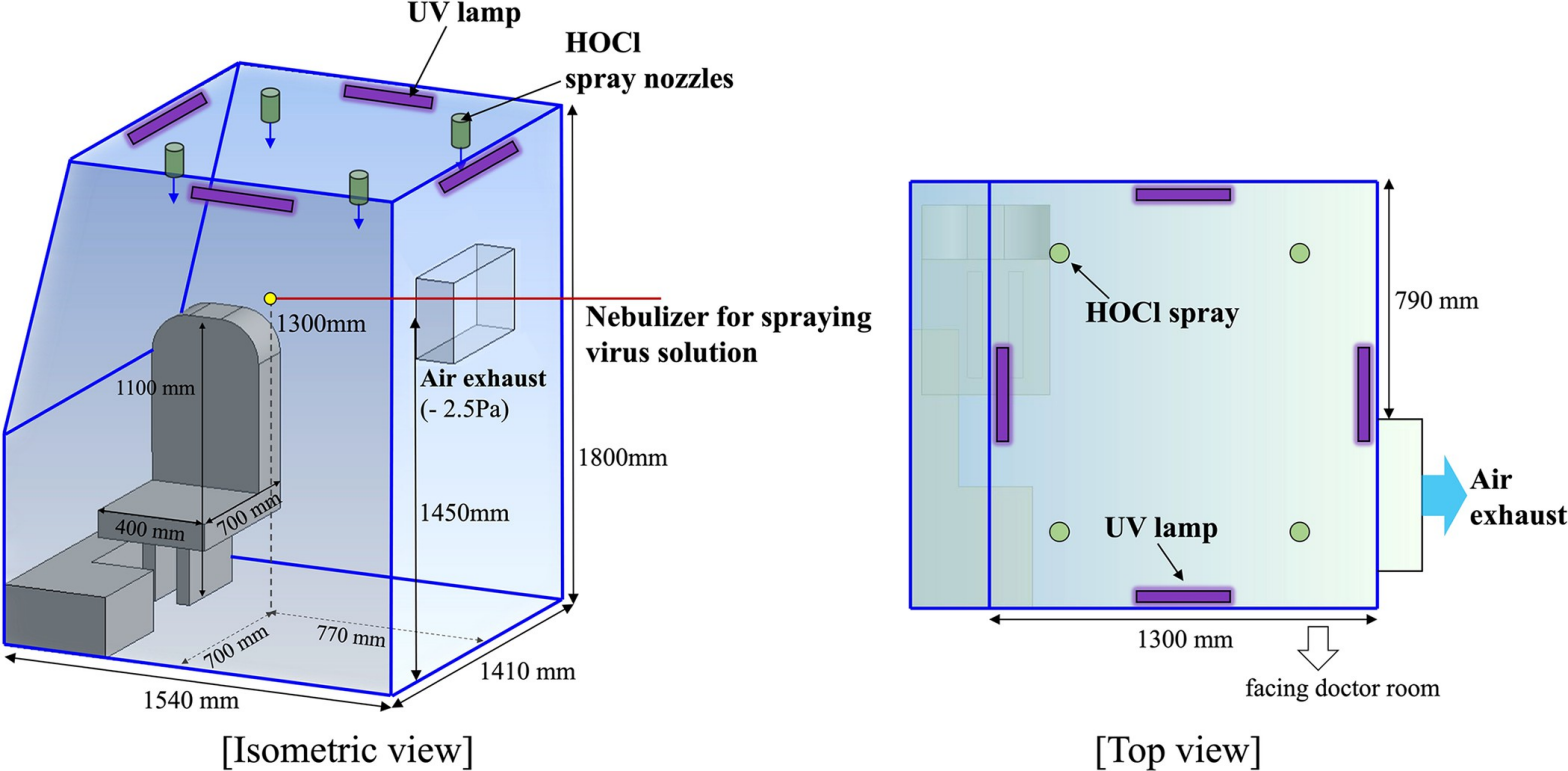
Schematics of the sterile chamber for infectious disease treatment (patient room in S1 Fig, for diagnostic testing). Each blue (solid line) edge denotes the air inlet in the form of a narrow gap in two wall joints.

The experiments were performed in a sterile chamber (1.5 × 1.4 × 1.8 m^3^) with one slanted side wall (Fig 1), which is the simplified model (dimensions are the same) of the patient compartment of the actual product that we have developed for fast massive testing against infectious airborne diseases with enhanced mobility (S1 Fig). That is, the present experiments for virus elimination were performed in the patient compartment separately from the real product. As shown in Fig 1, it was designed to be as compact as reasonably possible to be fit into the vehicle while also allowing the patient, if necessary, to enter the unit *via* the wheelchair. On the other hand, the patient room used in the real system has yellow latex gloves connecting from the doctor room to the patient room and the double-door entrance on the backside (S1 Fig). Since it was designed for a mobile medical unit that is installed (carried) in a large vehicle, the patient room has a single chair for the elderly and children, and various medical supplies are under the chair, including a refrigerator for specimen storage, thermometers, and sphygmomanometers. All of the disinfection equipment is operated from the controller located outside the chamber.

To evaluate the efficacy of negative-pressure ventilation, we installed three disinfection methods in the isolated unit: air exhaust equipment, UV lamps, and four HOCl spray nozzles for virus removal and inactivation (Fig 1). In order to faithfully reflect the mechanism of enhanced ventilation in a negative-pressure unit obtained from a previous numerical study [11] while considering the environment of in-vehicle installation, we installed the air exhaust on the upper part (1.45 m from the bottom) of the left side wall of the chamber. The exhaust has a rectangular cross-section (500 × 300 mm^2^) and always has maintained a negative pressure of -2.5 Pa inside the chamber. Then, we focused on ensuring that the patient’s respiratory flow with the pathogen-laden aerosol clusters was not dispersed. The air inlet, as a narrow gap, is located at two wall joints. As a UV irradiation, we considered the UV light with the wavelength of 253.7 nm (UV-C), which has been often used for sterilization [18], and the power of the lamp is 8 W. In total, four lamps are used, one at each of the four corners of the chamber ceiling. The 60 ppm HOCl (generated by electrolysis of 3% hydrochloric acid) spraying system consists of a pressure pump, a HOCl tank, and four spray nozzles that are installed on the ceiling in a square configuration. Each nozzle sprays the HOCl mist droplets (average size of 12.5 ± 1 μm) in a cone shape towards the floor, at a volume flux of 29 mL/min.

### Preparation and spraying of virus solutions

To accurately measure the virus elimination rate, we used the impaction method with air samplers and considered three active viruses: MS2, T3, and Phi-X174 (ATCC 15597-B1, 11303-B3, 13706-B1, respectively). These bacteriophage surrogates reduce health risks and serve as ethical options for monitoring pathogen transmission patterns in hospitals or other indoor spaces [19–22]. Furthermore, many studies have demonstrated that the surrogates we used exhibit similar responses to disinfection methods as SARS-CoV-2 [22]. Weyersberg *et al*. (2023) [22] observed that PhiX-174 is a safer and more suitable surrogate, as it shows a UV-C response more closely aligned with that of SARS-CoV-2. Similarly, Sultan *et al*. (2024) [23] utilized MS2 as a surrogate in studies observing SARS-CoV-2 reduction by triethylene glycol in typical office settings. The MS2 phage is considered an ideal surrogate for human viruses along with its prolonged environmental stability [24,25]. T3 phage, which demonstrated comparable removal rates and responses to disinfectants across all experimental cases in this study, is also a viable surrogate for SARS-CoV-2. In order to distribute and suspend these viruses in the air, virus solutions were produced. First, it is necessary to cultivate *Escherichia coli C* (*E*. *coli C*), the specific bacterium that serves as the host for the three bacteriophages (viruses) targeted in the present study. Next, 10–25 mL of Soybean-Casein Digest Broth (TSB) is poured into a 250-mL sterile triangle flask, and *E*. *coli C* is injected into the flask using the inoculation loop. It is then recommended to shake the flask at 225 ± 25 rpm while being incubated overnight at 35 ± 1°C. A 1-L triangular flask is to be filled with the dilution of the initial *E*. *coli C* culture and 100 mL of TSB in a ratio of 1:100. The *E*. *coli C* culture is incubated in the tri-flask at 35 ± 1°C for 18 ± 2 hours while being shaken at 225 ± 25 rpm. To preserve the culture, it is advised to store it at a temperature of 4 ± 1°C and utilize it within a span of 4 hours.

Next, we cultivate the cultures of three bacteriophages, which are the viruses that infect the bacteria. Three identical volumes of *E*. *coli C* culture are prepared following the above-mentioned instructions. In three triangle flasks containing the *E*. *coli C* culture, each of the three viruses (concentration of 1.0 × 10^9^–10^10^ pfu/mL; volume of 5 to 10 mL) is inoculated. The cultures that have been treated with an inoculum are subsequently stirred at a temperature of 35 ± 1°C for a duration of 1 to 5 hours, until the *E*. *coli C* cells undergo lysis. Cell lysis is considered complete when the cultures are exposed to 640 nm light and the absorbance ceases to decrease. The supernatant is transferred to a sterile test tube after centrifuging the culture for 20 minutes at 3000 rpm to get rid of any *E*. *coli C* debris. A 0.22-μm filter should be used to separate the supernatant. The virus cultures obtained usually have a concentration of 5.0 ± 2.0 × 10^10^ pfu/mL. It is advised to preserve these cultures at a temperature of 4 ± 1°C and utilize them within a month.

Lastly, the virus solutions are produced by diluting the virus culture ten times with saline (8.5 g of sodium chloride per liter) to achieve a concentration of 5 × 10^9^ pfu/mL. The virus solution, which has been prepared in advance, is introduced into a nebulizer and subsequently dispersed inside the test chamber through spraying.

### Exposure pathogens

To avoid infecting the personnel to perform the testing, we chose three bacteriophages, MS2, T3, and Phi-X174, as the viruses which infect the bacteria, to be dispersed in the chamber. We prepared the virus solutions for each and sprayed them into the chamber at a flow rate of 0.2 mL/min through a nebulizer (Devilbiss PulmoNeb, Compressor, Model 3655, USA) at 1.3 m above the floor. The mass median aerodynamic diameter (MMAD) of the generated virus droplet is 5 μm, which was set to target the size of exhaled aerosols that do not fall directly to the floor and faithfully follow the airflow [26–30]. It is expected that the droplets of this size eventually match the average size of virus-laden aerosols considered in our previous numerical study [11], as some liquid evaporates in less than a second, leaving the virus-laden droplet nuclei only with a size of about 1 μm [28,30]. The virus solution was sprayed for 10 minutes, and simultaneously, a stirring fan placed in the center of the floor was activated to distribute the virus aerosols uniformly in the chamber (see S2 Fig). At the end of the 10-minute homogenization period, the stirring fan was turned off.

### Acquisition of air sample and virus concentration

In this study, two viral collection media and air samplers are used to measure the initial and final virus concentrations following the disinfection treatment and ventilation in the chamber (S2 Fig). To accurately measure the virus elimination rate, we used the impaction method [31] with air samplers. Prior to each experiment, the virus-collecting media are prepared. First, combine 1.5 mL of *E*. *coli C* culture with 5 mL of top agar in a 15-mL test tube. The top agar consists of 99.3% TSB and 0.7% Bacto agar (Difco’s, 214010). Then, it is mixed promptly while taking care to avoid creating bubbles. Subsequently, this solution is carefully poured onto the bottom agar medium (Triptic soy agar, TSA) and mixed by spinning gently. Once the solution has solidified, it is flipped over and utilized as the medium for harvesting. If it is not utilized promptly, it is recommended that the media be stored at a temperature of 4 ± 1°C and consumed within 4 hours.

The prepared media are inserted into air samplers (Merck, MAS-100NT, USA), of which the suction pump takes in air (at the nominal flow rate of 100 L/min) and exposes it to the media. Prior to the experiment, the air sampler lid and cover undergo thorough sterilization using the autoclave. Additionally, they are disinfected with 70% alcohol or fully dehydrated if required. Virus concentration measurements are conducted at the front and back walls of the chamber, at a height of 0.9 meters above the floor (S2 Fig). At the end of each experiment, the final virus concentration is measured for 5 minutes, at which time all the disinfection devices and air exhaust are powered off.

### Procedure of experiment

Prior to conducting the experiment, it is essential to perform the necessary preliminary treatments. This includes adjusting the temperature and relative humidity of the air in the chamber to 23°C and 50%, respectively. Since the CDC suggests that the most suitable temperature and relative humidity in hospitals are 21–24°C and 30–60%, respectively, experimental environment was design to closely simulate the conditions of a hospital. The door of the chamber remains open, while the air within the room is regulated by an air purifier equipped with a HEPA filter for enhanced air purification, as well as an air conditioning system. The air purifier is also comprised of a primary filter and an activated carbon filter, which work together to eliminate ozone and odor within the test chamber. The pretreatment additionally guarantees that the viral concentration within the chamber is 0 pfu/m^3^.

After the pretreatment is finished, the chamber is subjected to a 10-minute nebulization process using a virus solution. Subsequently, a 40-minute interval is given to facilitate the stabilization of the virus-laden droplets within the chamber. Upon the end of the designated waiting period, two air samplers equipped with virus collection media are activated to quantify the initial concentration of virus both in the front and back walls of the chamber. Following the measurement of the initial concentration, natural decay tests (blank tests) are conducted to assess the natural decay rates, and operational decay tests are then performed using the measured data.

Natural decay tests are performed after a 40-minute waiting period with no ventilation equipment or disinfection devices in operation. After the initial concentration measurement, the chamber is allowed to stand for another 30 minutes without any external disturbance, and then the final concentration is measured. The process is repeated seven times to incubate the measured virus collection media, and then the colony count is performed to calculate the average initial (*c*_*i*_ [pfu/m^3^]) and final (*c*_*t*_ [pfu/m^3^]) concentrations. Therefore, the natural virus concentration decay rate is calculated as $B_{i}[\%]=\{\left(c_{i}-c_{t}\right)/c_{i}\}\times 100$, where *B*_*i*_ is the natural decay rate.

Operational decay test, as opposed to natural decay test, is the process of measuring the reduced virus concentration by operating disinfection or ventilation protocols. In this process, the initial concentration is corrected by *B*_*i*_, i.e., the initial concentration correction value (*S*_*c*_) is defined as $S_{c}[pfu/m^{3}]=\left(1-B_{i}\right)/100\times P_{t}$, where *P*_*t*_ [pfu/m^3^] is the initial virus concentration of operational decay test. The final virus concentration (*C*_*f*_ [pfu/m^3^]) is measured immediately after the end of the virus removal protocol determined in each experimental case. In this process, the measured media is incubated and then counted. The airborne virus elimination rate is thus defined as $R[\%]=\{\left(S_{c}-C_{f}\right)/S_{c}\}\times 100$.

### Incubation media and colony count

The media containing the airborne virus is gathered and cultured for 24 hours in an aerobic environment at a temperature range of 32–35°C. The quantity of plaque in the petri dish is enumerated following incubation. The media is carefully inspected to ensure that the quantity of colonies fells within the permissible range (≤ 300 pfu/petri plate) and to check for any contamination by external sources. The Feller table is utilized to adjust the value by incorporating the sample volume, resulting in the determination of the pfu value per unit volume.

## 3. Results

### First stage: Comparison of basic elements of virus removal methods

In the first stage of experiments, we measured the virus removal performance of ventilation, UV light, and HOCl spray individually. In this round, we ran each disinfection and ventilation step for the minimum duration required for sterilization, and Table 1 shows the ten different virus removal protocols that we conducted. For cases 1–3, each virus removal method was separately evaluated as 3 minutes of ventilation, 2 minutes of UV irradiation, and 15 seconds of HOCl spray, respectively. Ventilation and disinfection time for both HOCl and UV were determined based on achieving a reduction of over 90% of the virus while maintaining a concentration of chemical agents that would be safe for patients entering the unit. 3 minutes of ventilation time is based on our previous CFD study [11]. For UV, several previous studies have demonstrated that UV light can eliminate over 90% of viruses within 2 minutes [32,33]. Since the UV light is turned off when a patient enters the unit, potential UV exposure to the patient was not considered. For HOCl disinfection, there are no specific guidelines from the CDC or WHO regarding the amount of HOCl mist that may be harmful to humans. However, OSHA recommends that the concentration of chemical agents potentially harmful upon inhalation should be kept below 0.5 ppm in indoor environments (Permissible Exposure Limit, PEL). Therefore, we sprayed a 60 ppm HOCl solution for 15 seconds using four nozzles, resulting in an airborne HOCl concentration that remained below 0.5 ppm. In Table 1, the observation time refers to the time interval between the initial and final virus concentration measurements, and therefore, it varies from 3 to 20 minutes. Taking case 1 as an example, after the ventilation for 3 minutes, we waited for 17 minutes without any external disturbance before measuring the final virus concentration in the chamber. By doing this, the observation duration for cases 1–3 was matched to the actual clinical cases 9–10 for fair and accurate comparisons. Cases 9–10 are the protocols frequently used in hospital facilities; all three methods are combined, and the disinfection and ventilation activities are performed for 20 minutes. Cases 4–7 are different combinations of HOCl spray and ventilation. Because of the HOCl spraying process, we drastically reduced the ventilation duration to 45–90 seconds. Finally, the case 8 is the combination of UV light radiation and ventilation, which were performed for 2 and 3 minutes, respectively.

**Table 1:** The experimental cases evaluated in the first stage with the negative-pressure ventilation, UV (ultraviolet) light, and HOCl (Hypochlorite acid) spray. Each case consists of a combination of two distinct steps. The observation time encompasses not only the duration of the two steps, but also the period of waiting that follows the completion of the steps, unaffected by any external disturbances (for some cases, no waiting time is required).

	Step 1	Step 2	Observation	Elimination rate [%]		
				MS2	T3	Phi-X174
1	Ventilation 3 min	-	20 min	90.55	91.07	92.08
2	UV 2 min	-	20 min	97.09	93.78	93.99
3	HOCl spray 15 sec	-	20 min	6.16	6.97	4.76
4	Ventilation 45 sec	HOCl spray 15 sec	3 min	15.18	15.21	13.41
5	HOCl spray 15 sec	Ventilation 45 sec	3 min	21.13	21.07	17.57
6	Ventilation 1 min 30 sec	HOCl spray 15 sec	3 min	18.72	21.09	19.32
7	HOCl spray 15 sec	Ventilation 1 min 30 sec	3 min	25.81	23.72	24.46
8	UV 2 min	Ventilation 3 min	5 min	98.92	97.12	98.62
9	Ventilation 10 min	HOCl spray + UV 10 min	20 min	99.90	99.99	99.96
10	HOCl spray + UV 10 min	Ventilation 10 min	20 min	99.92	99.99	99.97

**Fig 2:**
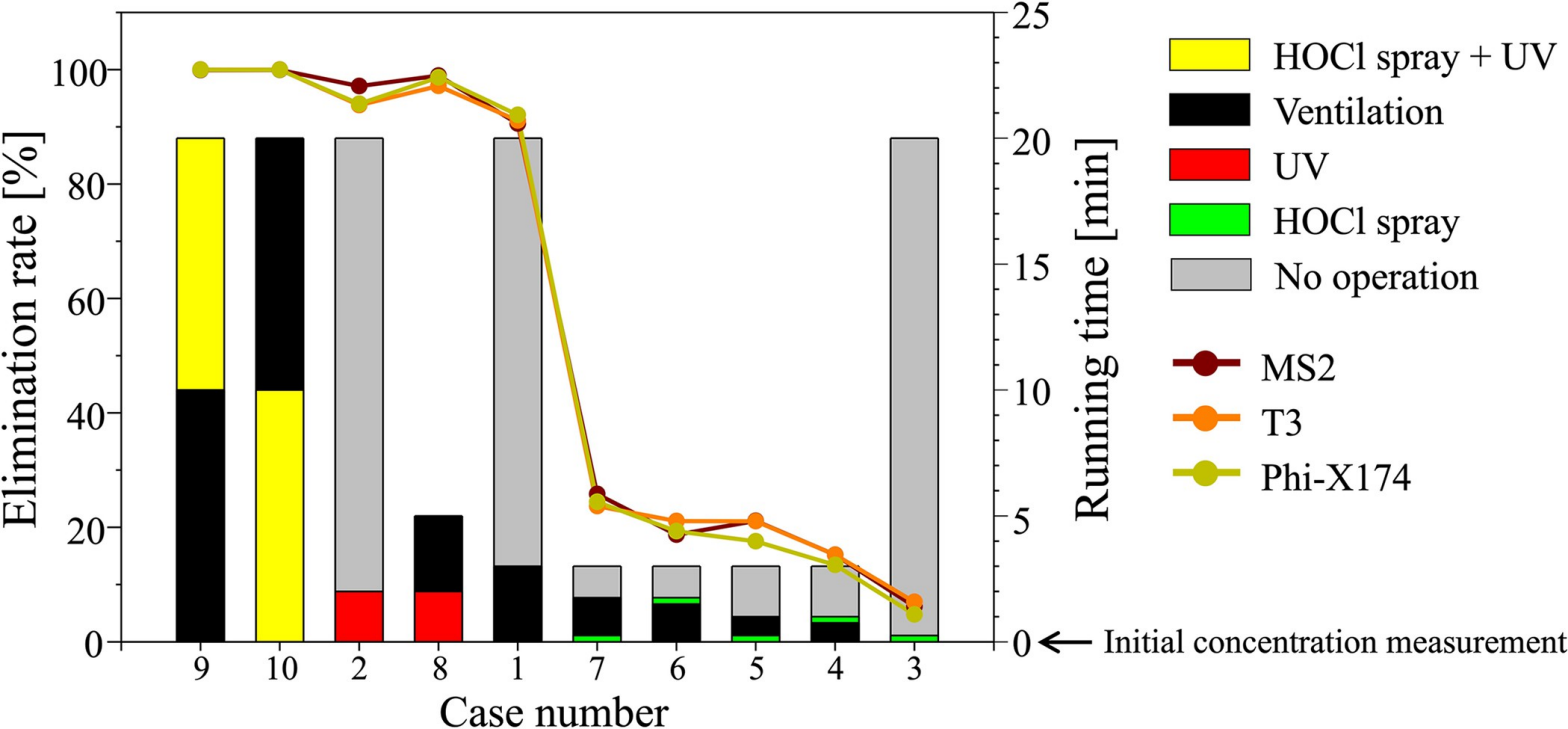
Virus removal rate (line-scatter profiles) and protocol running time (bar graphs) for all the cases tested in the first stage of the experiment. The initial virus concentration is measured at running time 0. The bar graphs are displayed chronologically, illustrating the sequence of steps done in the protocol over time.

Fig 2 shows the three virus elimination rates (line-scatter profiles) and the running time (bar graphs) of the ten virus removal protocols during the observation time. Unsurprisingly, cases 9–10 show a 99.9% elimination rate of all three viruses when all three processes were applied fully for 20 minutes of present observation time. Comparing the other cases, it is found that the HOCl spray disinfection method has a significantly lower performance for virus inactivation than ventilation or UV light irradiation. When each virus removal method is applied alone, the ventilation (case 1) and UV light (case 2) showed more than 90% of the virus elimination in the chamber, while the elimination rate by the HOCl spray (case 3) is below 10%. This trend is also found for cases 4–7 including the HOCl spray; the virus elimination rate is still less than 30% due to the relatively short ventilation duration. Among them, the elimination rate increases when the ventilation duration is longer (cases 6 and 7). This clearly indicates that properly designed ventilation is quite effective in removing the virus. The next noticeable point learned from the first stage of the experiment is that the performance of UV light (94.95%) is as good as ventilation (91.23%). Indeed, with case 8 (a combination of UV light and ventilation), we obtained a virus elimination rate as high as 98%, despite the short (5 minutes) time for applying the protocols. This is comparable to the performance of conventional long-duration (20 minutes) protocols (cases 9–10), which are inefficient based on our recent experiences with worldwide pandemic situations.

### Second stage: Optimization of the hybrid protocols

In the second stage of the experiment, we focused on how to optimize the hybrid protocol. Since the elimination rates of the three-virus species in the first stage were similar in all cases, we only considered T3 as the target virus in the second round. As shown in Table 2, we designated the observation duration from 2 to 7 minutes in 1-minute increments, listing 14 combinations for UV light only, simultaneous UV light irradiation, and ventilation, and the simultaneous UV light and ventilation followed by the additional ventilation.

**Table 2:** The experimental cases evaluated in the second stage with negative-pressure ventilation and UV (ultraviolet) light. Each case consists of a combination of two distinct steps. The observation time encompasses not only the duration of the two steps, but also the period of waiting that follows the completion of the steps, unaffected by any external disturbances (in some cases, no waiting time is required).

	Step 1	Step 2	Observation	Elimination rate [%]
				T3
1	UV 2 min	-	2 min	98.6
2	UV + Ventilation 2 min	-	3 min	97.2
3	UV + Ventilation 3 min	-	3 min	97.4
4	UV + Ventilation 2 min	Ventilation 1 min	3 min	98.2
5	UV 3 min	-	3 min	99.2
6	UV + Ventilation 2 min	Ventilation 2 min	4 min	98.1
7	UV 4 min	-	4 min	99.0
8	UV + Ventilation 3 min	Ventilation 1 min	4 min	99.6
9	UV + Ventilation 2 min	Ventilation 3 min	5 min	98.8
10	UV 5 min	-	5 min	99.0
11	UV + Ventilation 3 min	Ventilation 2 min	5 min	99.5
12	UV + Ventilation 3 min	Ventilation 3 min	6 min	99.2
13	UV 6 min	-	6 min	99.9
14	UV 7 min	-	7 min	99.9

**Fig 3:**
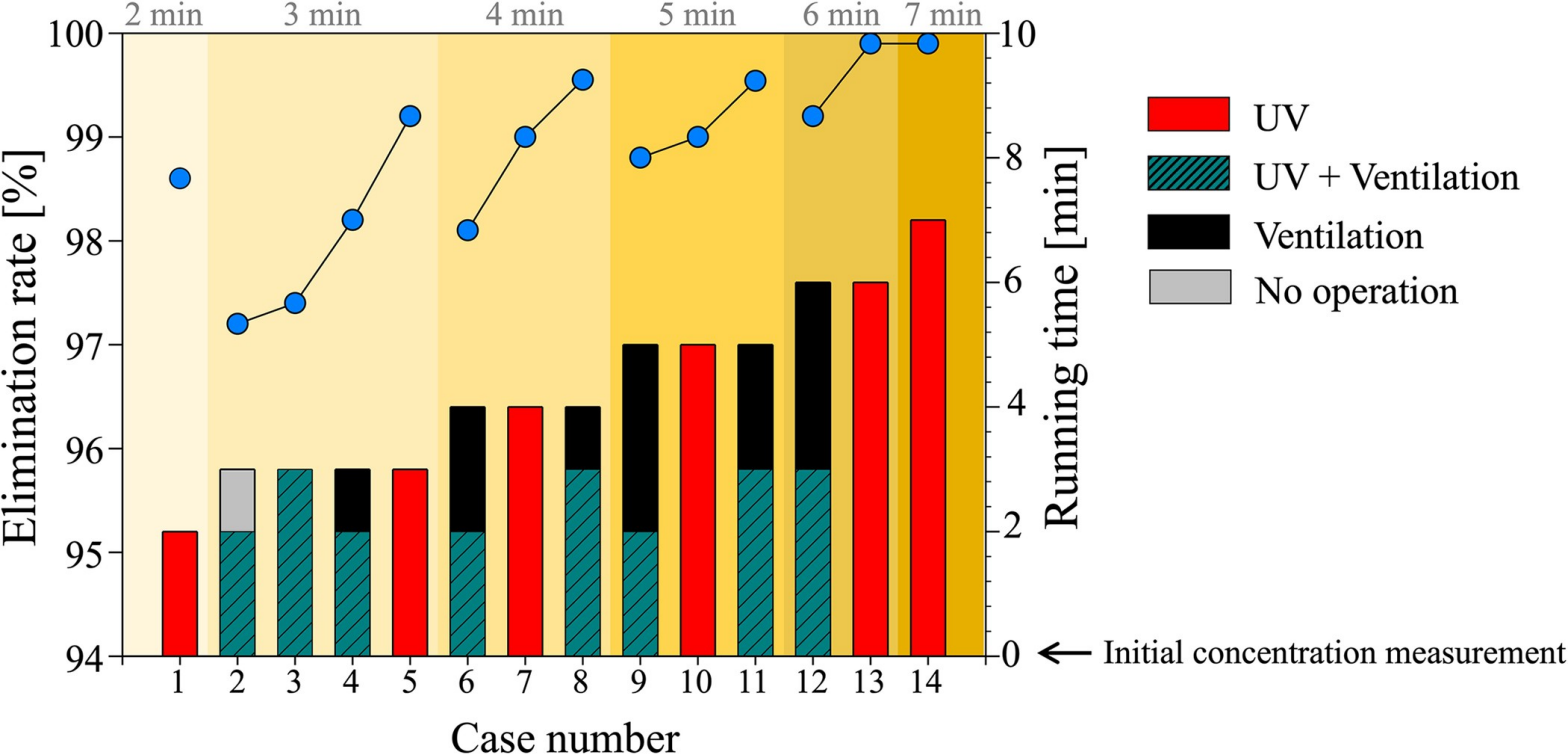
Virus removal rate (line-scatter profiles) and protocol running time (bar graphs) for all the cases tested in the second stage of the experiment. The initial virus concentration is measured at running time 0. The bar graphs are displayed chronologically, illustrating the sequence of steps done in the protocol over time.

The virus elimination rate (line-scatter profiles) and protocol running time (bar graphs) of the cases in Table 2 are compared in Fig 3. The viricidal performance of both UV light and ventilation is so excellent that the elimination rate is over 97% in all cases. Since our ultimate goal is to identify (i.e., optimize) the protocol that eliminates more than 99% of viruses in the shortest time duration, we focused our analysis on the cases with a 99% or higher elimination rate. As a result, it is noted that it is not necessarily mandatory to run the UV light and ventilation for a longer duration (> 5 minutes), but it is still sufficient to run the virus removal protocol for only 3–4 minutes (cases 5 and 7 with UV irradiation for 3 and 4 minutes, respectively, and case 8 with simultaneous UV light and ventilation for 3 minutes followed by the additional 1-minute ventilation) to achieve a virus elimination rate of over 99%.

## 4. Discussion

The present results suggest virucidal methodologies and protocols that achieve 99% of the active virus being eliminated inside the isolation unit. To the best of our knowledge, this is the first report that no one has proven experimentally. In clinical environments to date, 10 minutes of negative-pressure ventilation (at -2.5 Pa) and 10 minutes of HOCl spray + UV irradiation have been used after patients have left the room in infectious disease specimen collection booths or clinics as the indoor airborne virucidal methods; these are the same as cases 9 and 10 of the first stage experiment in this study. However, there has never been a consensus for these virus removal protocols to stick in hospital environments, implicating that while many medical studies have individually validated the sterilization capabilities of negative-pressure ventilation, chemical virucidal liquid agents, and UV irradiation, the combination of them has never been clinically evaluated. First, we set the minimum requirement of time to apply each methodology to eliminate the viruses based on previous studies and compared the three methodologies against each other. The ventilation time for three minutes in this study was determined based on the results of our previous research [11], in which the air exhaust can discharge 99% of pathogens out of the chamber in five minutes. It was reduced to three minutes, which is the minimum time clinically required for a medical staff to process the patient specimens or prepare for treatment. This is because we intended to assess how much virus can be eliminated with the least amount of negative-pressure ventilation within the isolation unit. Additionally, the HOCl solution and UV irradiation have been clinically evaluated as viricidal agents.

However, these two methodologies have been evaluated in a variety of non-standardized experimental setups, resulting in inconsistent results across the literature. In the case of HOCl, Tazawa *et al*. [15] reported that the time required for a sprayed HOCl solution obtained by 3% HOCl electrolysis to inactivate the microorganisms was 30 seconds. Hakim *et al*. [34] reported that the 5–10 second spray of 100–200 ppm HOCl solution can reduce the concentration of avian influenza virus (AIV) to an undetectable level. Compromising the results of previous studies, we set the HOCl solution spraying duration in this study to 15 seconds, and it was measured that its performance (case 3) is below 10% in killing the virus. Thus, it is judged that even if the duration is extended, it is unlikely to be more effective than UV light or ventilation. For the UV irradiation, Heilingloh *et al*. [35] reduced the SARS-CoV-2 infectivity to nearly 0% with a 3-minute UV-C (1.94 mW/cm^2^) and UV-A (0.54 mW/cm^2^) treatment combination, while Kitagawa *et al*. [32] inactivated more than 99% of SARS-CoV-2 within 30 seconds with 222 nm UV (0.1 mW/cm^2^) irradiation. This scattering would be affected by variations in virus concentration, distance between the UV light and the virus plate, and UV light power. In other words, as with HOCl spraying, there are no specific guidelines or regulations for the clinical usage of UV light irradiation. Therefore, we decided to apply the UV light (0.18 W/cm^2^) at 253.7 nm (UV-C), which showed the maximum microbicidal activity^11^, for 2 minutes in the first stage tests. Through this process, we confirmed that UV light has the highest virus inactivation efficacy, followed by negative-pressure ventilation and HOCl spray in the first-stage experiments.

Subsequently, the combination of UV irradiation and negative-pressure ventilation was considered to be the candidate for optimal protocols, and we evaluated it in the second stage experiments. Given the high effectiveness of UV sterilization, it is more efficient to use UV in conjunction with the ventilation rather than running the ventilation system alone for more than 10 minutes. Our clinical interpretations of the results implicate that (i) the UV light alone for three minutes (case 5) and (ii) the UV light with ventilation for three minutes, followed by the one-minute ventilation only–hybrid protocols (case 8) are the most effective virucidal protocols. This is because of the consideration that it is dangerous for people to enter the unit when the UV radiation still remains (e.g., case 7 of UV light for four minutes). In case 8, the simultaneous operation of UV light and ventilation is followed by one minute of ventilation, which allows the doctors or patients to check in advance and prepare for the next treatment, which will also help speed up patient circulation in the facility.

On the other hand, there are a few limitations to this study. First, laboratory bacteriophages were used in the experiments, and there is a restriction that real clinical viruses were not evaluated. Therefore, it would not be readily possible to completely respond to the spread of a novel virus with unexpectedly high infectiousness and transmissibility. While this is considered an unavoidable situation in the preparation of newly emerging diseases, the present optimal protocols based on rigorous scientific grounds would perform better than the present ones based on experience and tradition. Second, the concentration of virus within the space may change as the surface area of the diagnostic facility varies, which would affect the test results. As explained above, however, the present experiments were conducted based on the size of a typical diagnostic facility, including our previous study. Lastly, the inactivation susceptibility of each respiratory virus to UV light is known to be different. When a novel respiratory virus emerges in the future, it will be necessary to evaluate the effectiveness of UV light and HOCI spray as disinfection methods.

## 5. Conclusion

In summary, we recommend two optimal virus removal protocols in an isolation unit (hospital environment): (i) UV light alone for 3 minutes and (ii) a combination of UV light and ventilation for 3 minutes followed by 1-minute ventilation. For practical application in a clinical environment, 3-minute UV irradiation would be more favorable among the two protocols because it can be realized with the most compact installation of disinfection devices inside the room. Beyond other studies that have clinically demonstrated the superiority of UVC disinfection, the present study provides the exact number of minutes of UV irradiation required. We firmly believe that these protocols optimized based on physical and practical grounds are the most clinically reasonable virus removal methodology in the isolation unit because they satisfy the minimum time required for the medical staff to reorganize the chamber safely while achieving a viral kill rate of over 99%. If recommended disinfection and ventilation protocols are implemented as a public policy as we wish eagerly, the massive testing expected during the next pandemic can be carried out effectively. It is expected to contribute to maximizing efficiency by shortening virus removal time, and increasing the patient turnover at diagnostic facilities within hospitals or mobile diagnostic unit outside of hospital.

## Supporting information

S1 Fig Illustration of the mobile medical unit installed in a vehicle designed to treat the infectious diseases with a positive pressure doctor room and a negative pressure patient room. (a) vehicle with the unit installed; (b) vehicle interior schematic (various sterilization equipment and experimental tools not shown); (c) wheelchair rails installed on the vehicle, and latex gloves on the chamber wall.
(JPG)

S2 Fig Illustration of the isolated patient room with experimental tools such as air samplers (with media) and a stirring fan. The stirring fan is placed in the center of the floor. Two virus collection media and air samplers are at the front and back walls of the chamber, at a height of 0.9 meters above the floor. Note that various sterilization equipment is excluded in this figure.
(JPG)

## References

[1] Qian H, Li Y. Removal of exhaled particles by ventilation and deposition in a multibed airborne infection isolation room. *Indoor Air* 2010; 20:284–97. doi: 10.1111/j.1600-0668.2010.00653.x 20546037

[2] Yang R, Ng CS, Chong KL, Verzicco R, Lohse D. Do increased flow rates in displacement ventilation always lead to better results? *J*. *Fluid Mech*. 2020; 932:A3. doi: 10.1017/jfm.2021.949

[3] Weiland N, Traversari R, Sinnige T, van Someren Gréve F, Timmermans A, Spijkerman I, et al. Influence of room ventilation settings on aerosol clearance and distribution. *Br*. *J*. *Anaesth* 2021; 126:e49–52. doi: 10.1016/j.bja.2020.10.018 33190858 PMC7584416

[4] Liu H, He S, Shen L, Hong J Simulation-bassed study of COVID-19 outbreak associated with air-conditioning in a restaurant. *Phys*. *Fluids* 2021; 33:023301. doi: 10.1063/5.0040188 33746488 PMC7976041

[5] Traversari AAL, van Heumen SPM, van Tiem FLJ, Bottenheft C, Hinkerma MJ. Design variables with significant effect on system performance of unidirectional displacement airflow systems in hospitals. *J*. *Hosp*. *Infect*. 2019; 103:e81–7. doi: 10.1016/j.jhin.2019.03.009 30923013

[6] Centers for Disease Control. *Guidelines for environmental infection control in health-care facilities*. 2003. www.cdc.gov.

[7] Dinoi A, Feltracco M, Chirizzi D, Trabucco S, Conte M, Gregoris E, et al. A review on measurements of SARS-CoV-2 genetic material in air in outdoor and indoor environments: implication for airborne transmission. *Sci*. *Total Environ*. 2022; 809:151137. doi: 10.1016/j.scitotenv.2021.151137 34699823 PMC8539199

[8] Correia G, Rodrigues L, Da Silva MG, Gonçalves T. Airborne route and bad use of ventilation systems as non-negligible factors in SARS-CoV-2 transmission. *Med*. *Hypotheses* 2020; 141:109781. doi: 10.1016/j.mehy.2020.109781 32361528 PMC7182754

[9] Hadei M, Hopke PK, Jonidi A, Shahsavani A. A Letter about the airborne transmission of SARS-CoV-2 based on the current evidence. *AAQR* 2020; 20:911–4. doi: 10.4209/aaqr.2020.04.0158

[10] Liu Y, Ning Z, Chen Y, Guo M, Liu Y, Gali N, et al. Aerodynamic analysis of SARS-CoV-2 in two Wuhan hospitals. *Nature* 2020; 582:557–60. doi: 10.1038/s41586-020-2271-3 32340022

[11] Park J, Lee KS, Park H. Optimized mechanism for fast removal of infectious pathogen-laden aerosols in the negative-pressure unit. *J*. *Hazard*. *Mater*. 2022; 435:128978. doi: 10.1016/j.jhazmat.2022.128978 35472540 PMC9020843

[12] Bhagat RK, Wykes MD, Dalziel SB, Linden P. Effects of ventilation on the indoor spread of COVID-19. *J*. *Fluid Mech*. 2020; 903:F1. doi: 10.1017/jfm.2020.720 34191877 PMC7520710

[13] Sodiq A, Khan MA, Nass M, Amhamed A. Addressing COVID-19 contagion through the HVAC systems by reviewing indoor airborne nature of infectious microbes: will an innovative air recirculation concept provide a practival solution? *Environ*. *Res*. 2021; 119:11329. doi: 10.1016/j.envres.2021.111329 34004171 PMC8123526

[14] Villafruela JM, Olmedo I, Berlanga FA, Ruiz de Adana M. Assessment of displacement ventilation systems in airborne infection risk in hospital rooms. *PLoS One* 2019; 14:e0211390. doi: 10.1371/journal.pone.0211390 30699182 PMC6353581

[15] Tazawa K, Jadhav R, Azuma MM, Fenno JC, McDonald NJ, Sasaki H. Hypochlorous acid inactivates oral pathogens and a SARS-CoV-2-surrogate. *BMC Oral Health* 2023; 23:111. doi: 10.1186/s12903-023-02820-7 36803460 PMC9938691

[16] Rutala WA, Weber DJ. Disinfection, Sterilization, and antisepsis: an overview. *Am*. *J*. *Infect*. *Control* 2019; 47:A3–9. doi: 10.1016/j.ajic.2019.01.018 31146848

[17] Kompatscher K, van der Vossen JMBM, van Heumen SPM, Traversari AAL. Scoping review on the efficacy of filter and germicidal technologies for capture and inactivation of micro-organisms and viruses. *J*. *Hosp*. *Infect*. 2023; 142:39–48. doi: 10.1016/j.jhin.2023.08.026 37797657

[18] Russell AD, Hugo WB, Ayliffe GAJ. *Principles and practice of disinfection*, *preservation and sterilization*. *Oxford*: *Blackwell Science*, 1999.

[19] Sassi HP, Sifuentes LY, Koenig DW, Nichols E, Clark-Greuel J, and Wong LF, et al. Control of the spread of viruses in a long-term care facility using hygiene protocols. *Am*. *J*. *Infect*. *Contr*. 2015; 43:702–6. doi: 10.1016/j.ajic.2015.03.012 25944726

[20] Sifuentes LY, Koenig DW, Phillips RL, Reynolds KA, Gerba CP. Use of hygiene protocols to control the spread of viruses in a hotel. *Food Environ*. *Virol*. 2014; 6:175–81. doi: 10.1007/s12560-014-9158-0 25005587

[21] Valdez MK, Sexton JD, Lutz EA, Reynolds KA. Spread of infectious microbes during emergency medical response. *Am*. *J*. *Infect*. *Contr*. 2015; 43:606–11. doi: 10.1016/j.ajic.2015.02.025 26042849 PMC7115268

[22] Weyersberg L, Sommerfeld F, Vatter P, Hessling M. UV radiation sensitivity of bacteriophage PhiX174 –A potential surrogate for SARS-CoV-2 in terms of radiation inactivation. *AIMS Microbiol*. 2023; 9:431–43. doi: 10.3934/microbiol.2023023 37649795 PMC10462461

[23] Sultan Z, Luhung I, Aung NW, Uchida A, Natarajan A, and Puramadathil S, et al. Effectiveness of thiethylene glycol disinfection on airborne MS2 bacteriophage under diverse building operational parameters. *Indoor Environ*. 2024; 1:100042. doi: 10.1016/j.indenv.2024.100042

[24] Bae J, Schwab KJ. Evaluation of murine norovirus, feline calicivirus, poliovirus, and MS2 as surrogates for human norovirus in a model of viral persistence in surface water and groundwater. *Appl*. *Environ*. *Microbiol*. 2008; 74: 477–84. doi: 10.1128/AEM.02095-06 18065626 PMC2223264

[25] Julian TR, Leckie JO, Boehm AB. Virus transfer between fingerpads and fomites. *J*. *Appl*. *Microbiol*. 2010; 109:1868–74. doi: 10.1111/j.1365-2672.2010.04814.x 20659186

[26] World Health Organization. *Infection Prevention and Control of Epidemic- and Pandemic-Prone Acute Respiratory Infections in Health Care*. 2014. www.who.int. 24983124

[27] Bake B, Larsson P, Ljungkvist G, Ljungström E, Olin AC. Exhaled particles and small airways. *Respir*. *Res*. 2019; 20:8. doi: 10.1186/s12931-019-0970-9 30634967 PMC6330423

[28] Fennelly KP. Particle sizes of infectious aerosols: implications for infection control. *Lancet Respir*. *Med*. 2020; 8: 914–24. doi: 10.1016/S2213-2600(20)30323-4 32717211 PMC7380927

[29] Milton DK. A rosetta stone for understanding infectious drops and aerosols. *J*. *Pediat*. *Inf*. *Dis*. 2020; 9:413–5. doi: 10.1093/jpids/piaa079 32706376 PMC7495905

[30] Fabian P, McDevitt JJ, DeHaan WH, Fung ROP, Cowling BJ, Chan KH. et al. Influenza virus in human exhaled breath: an observational study. *PLoS One* 2008; 3:e2691. doi: 10.1371/journal.pone.0002691 18628983 PMC2442192

[31] Lee JH, Kim JY, Cho B, Anusha JR, Sim JY, Raj CJ, et al. Assessment of air purifier on efficient removal of airborne bacteria, Staphylococcus epidermidis, using single-chamber method. *Environ*. *Monit*. *Assess*. 2019; 191:720. doi: 10.1007/s10661-019-7876-3 31691038 PMC7087645

[32] Kitagawa H, Nomura T, Nazmul T, Omori K, Shigemoto N, and Sakaguchi T, et al. Effectiveness of 222-nm ultraviolet light on disinfecting SARS-CoV-2 surface contamination. *Am*. *J*. *Infect*. *Control* 2021; 49:299–301. doi: 10.1016/j.ajic.2020.08.022 32896604 PMC7473342

[33] Rathnasinghe R, Karlicek RF, Schotsaert M, Koffas M, Arduini BL, and Jangra S, et al. Scalable, effective, and rapid decontamination of SARS-CoV-2 contaminated N95 respirators using germicidal ultraviolet C (UVC) irradiation device. *Sci*. *Rep*. 2021; 11:19970. doi: 10.1038/s41598-021-99431-5 34620951 PMC8497543

[34] Hakim H, Thammakarn C, Suguro A, Ishida Y, Kawamura A, Tamura M, et al. Evaluation of sprayed hypochlorous acid solutions for their virucidal activity against avian influenza virus through in vitro experiments. *J*. *Vet*. *Med*. *Sci*. 2015; 77:211–5. doi: 10.1292/jvms.14-0413 25421399 PMC4363024

[35] Heilingloh CS, Aufderhorst UW, Schipper L, Dittmer U, Witzke O, Yang D, et al. Susceptibility of SARS-CoC-2 to UV irradiation. *Am*. *J*. *Infect*. *Control*. 2020; 48:1273–5. doi: 10.1016/j.ajic.2020.07.03 32763344 PMC7402275

