# Supplementary figures and images for: Development of optimal indoor air disinfection and ventilation protocols for airborne infectious diseases
Source: PLoS One. 2024 Oct 1;19(10):e0311274. doi: 10.1371/journal.pone.0311274 (PMC11444385; doi:10.1371/journal.pone.0311274)

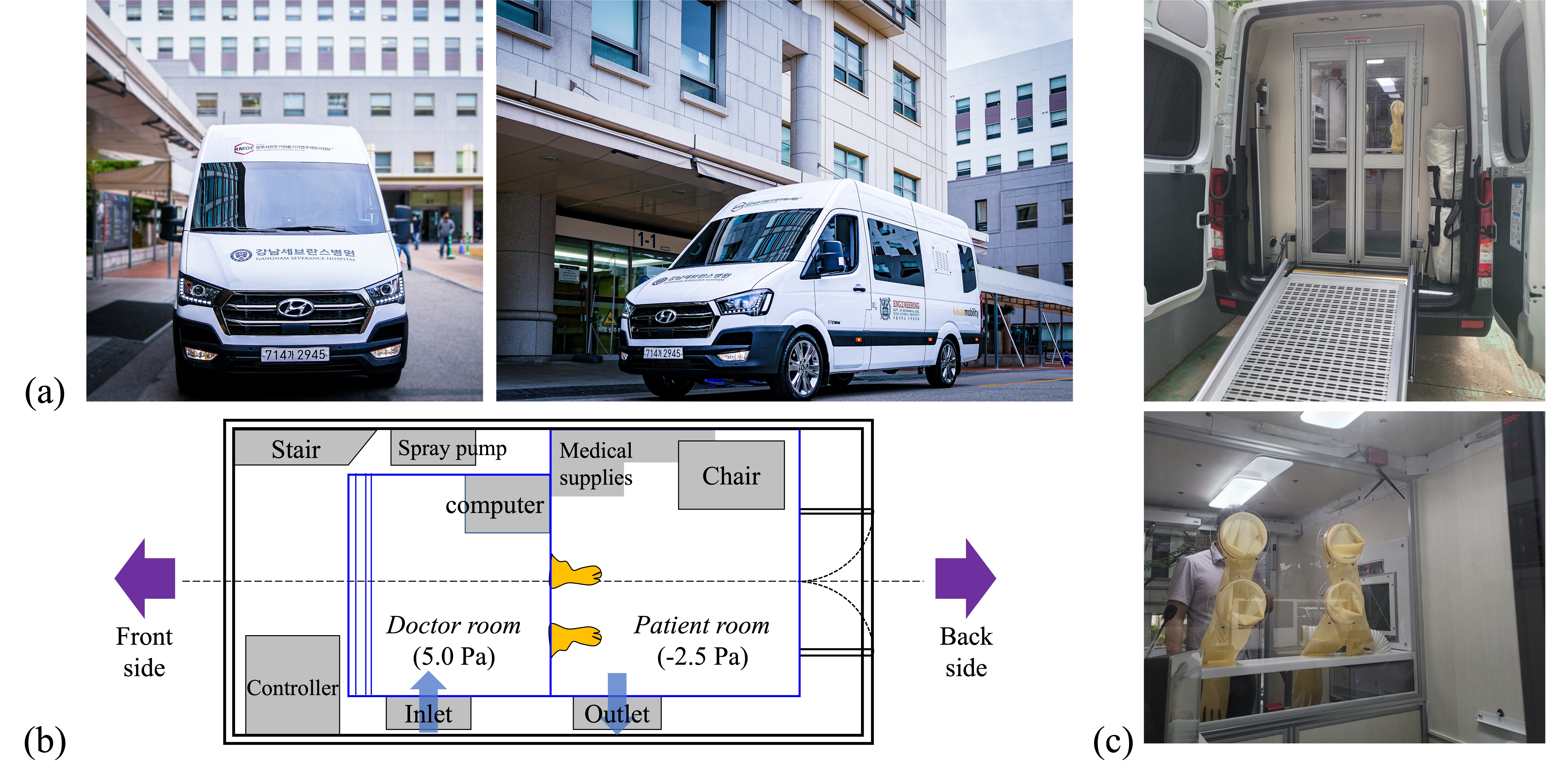

Supplement: S1 Fig — (a) vehicle with the unit installed; (b) vehicle interior schematic (various sterilization equipment and experimental tools not shown); (c) wheelchair rails installed on the vehicle, and latex gloves on the chamber wall. (JPG) [file pone.0311274.s001.jpg]

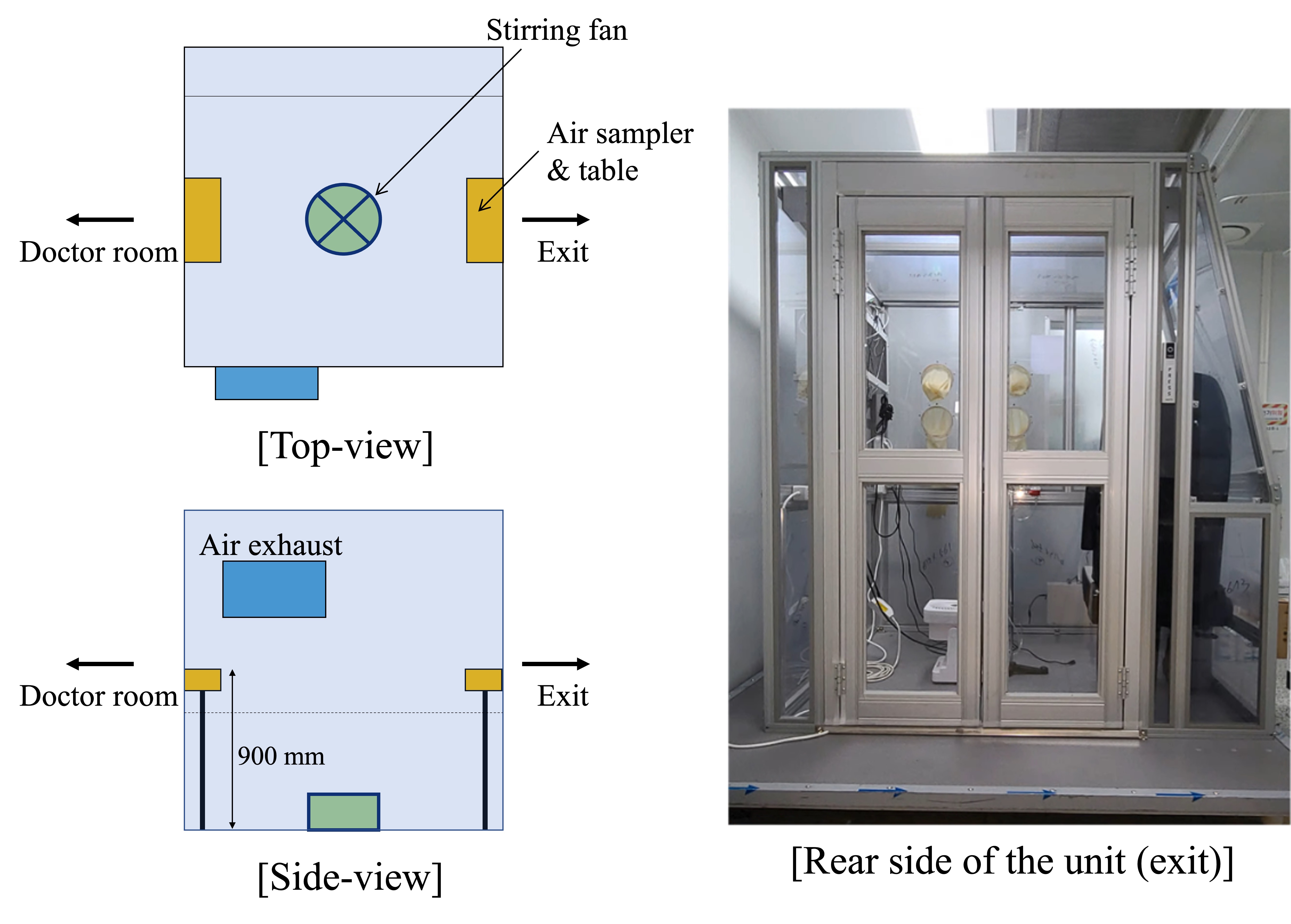

Supplement: S2 Fig — The stirring fan is placed in the center of the floor. Two virus collection media and air samplers are at the front and back walls of the chamber, at a height of 0.9 meters above the floor. Note that various sterilization equipment is excluded in this figure. (JPG) [file pone.0311274.s002.jpg]
